# Physical, Chemical, Barrier, and Antioxidant Properties of Pectin/Collagen Hydrogel-Based Films Enriched with *Melissa officinalis*

**DOI:** 10.3390/gels9070511

**Published:** 2023-06-25

**Authors:** Saurabh Bhatia, Ahmed Al-Harrasi, Aysha Salim Alhadhrami, Yasir Abbas Shah, Sabna Kotta, Javed Iqbal, Md Khalid Anwer, Anjana Karunakaran Nair, Esra Koca, Levent Yurdaer Aydemir

**Affiliations:** 1 Natural and Medical Sciences Research Center, University of Nizwa, Birkat Al Mauz, P.O. Box 33, Nizwa 616, Oman; ayshalhadhrami@gmail.com (A.S.A.); yasir.shah@unizwa.edu.om (Y.A.S.); 2 School of Health Science, University of Petroleum and Energy Studies, Dehradun 248007, India; 3 Saveetha Institute of Medical and Technical Sciences, Saveetha University, Chennai 600077, India; 4 Department of Pharmaceutics, Faculty of Pharmacy, King Abdulaziz University, Jeddah 21589, Saudi Arabia; skotta@kau.edu.sa; 5 Center of Excellence for Drug Research and Pharmaceutical Industries, King Abdulaziz University, Jeddah 21589, Saudi Arabia; 6 Center of Nanotechnology, King Abdulaziz University, Jeddah 21589, Saudi Arabia; iqbaljavedch@gmail.com; 7 Department of Pharmaceutics, College of Pharmacy, Prince Sattam Bin Abdulaziz University, Al-Kharj 11942, Saudi Arabia; m.anwer@psau.edu.sa; 8 Department of Biomedical Sciences, College of Pharmacy, Shaqra University, Shaqra 11961, Saudi Arabia; anju@su.edu.sa; 9 Department of Food Engineering, Faculty of Engineering, Adana Alparslan Turkes Science and Technology University, Adana 01250, Turkey; esrakoca.tr@outlook.com (E.K.); lyaydemir@atu.edu.tr (L.Y.A.)

**Keywords:** biopolymers, edible films, composite material, food packaging, essential oil

## Abstract

The essential oil extracted from *Melissa officinalis* (MOEO) exhibits a wide range of therapeutic properties, including antioxidant, antibacterial, and antifungal activities. The current research aimed to analyze the mechanical, barrier, chemical, and antioxidant properties of pectin and collagen-based films. Hydrogel-based films loaded with varying concentrations of MOEO (0.1%, 0.15%, and 0.2%) were prepared by solvent-casting method, and their physicochemical as well as antioxidant properties were examined. GC-MS analysis revealed the presence of major components in MOEO such as 2,6-octadienal, 3,7-dimethyl, citral, caryophyllene, geranyl acetate, caryophyllene oxide, citronellal, and linalool. Fourier transform infrared (FTIR) results revealed the interaction between components of the essential oil and polymer matrix. Scanning electron microscopy (SEM) revealed that films loaded with the highest concentration (0.2%) of MOEO showed more homogeneous structure with fewer particles, cracks, and pores as compared to control film sample. MOEO-incorporated films exhibited higher elongation at break (EAB) (30.24–36.29%) and thickness (0.068–0.073 mm); however, they displayed lower tensile strength (TS) (3.48–1.25 MPa) and transparency (87.30–82.80%). MOEO-loaded films demonstrated superior barrier properties against water vapors. According to the results, the incorporation of MOEO into pectin–collagen composite hydrogel-based films resulted in higher antioxidant properties, indicating that MOEO has the potential to be used in active food packaging material for potential applications.

## 1. Introduction

The food packaging material in the form of polymeric edible films is gaining more attention these days due to its numerous benefits, such as being edible, biodegradable, nontoxic, biocompatible, etc. These films are made at a laboratory scale to improve the safety, quality, and shelf life of food products [1]. There are several manufacturers (Innoteq, NewGem Foods, Watson, Biofilm Limited, ODF Pharma, Proinec, MonoSol, Umang Pharmatech, etc.) that make edible films at large scale. Pectin, a major constituent of plant cells, is primarily used in the manufacturing of fruit juices, jams, and bread fillings [2,3]. The utilization of pectin as a polymer to produce hydrogel-based films is a viable option owing to its gelation characteristics, lack of toxicity, biodegradability, and accessibility. Collagen is one of the natural polymers that hold significant potential as a vital industrial raw material [4]. Its physicochemical characteristics, biodegradability, and non-toxicity have made it increasingly important and useful in the food sector [5].

Several research studies have shown that incorporating various plant-based bioactive components into pectin-based films can result in to effective food packaging material [6,7]. Blending two polymers (pectin/collagen) is an effective way to synthesize the edible film with favorable barrier and mechanical attributes. Hydrogel-based films have been formulated with essential oils due to their possible antibacterial, antioxidant, and antifungal characteristics [8]. Essential oils are widely used in the food industries due to their composition of volatile compounds, including a combination of terpenes, terpenoids, alpha-aromatic and aromatic compounds, as well as non-volatile compounds [9].

*Melissa officinalis* is a member of the Lamiaceae family and possesses diverse therapeutic properties, and Melissa oil exhibits antibacterial, antifungal, and antioxidant properties [10,11]. Melissa essential oil (MOEO) primarily comprises citronella, but it also contains other components such as citral, geraniol, linalool, and beta-caryophyllene [12]. In earlier research, researchers developed active films using a combination of carboxymethyl chitosan and locust bean gum. These films contained nanoemulsions of essential oil extracted from *Melissa officinalis* L. Following the analysis of the results, it was noted that incorporating MOEO nanoemulsion into the active films resulted in an elevation of their elasticity and resistance to water [13]. Another research revealed the development of active edible films using sodium caseinate, which contained a combination of zinc oxide nanoparticles (ZnONPs) and microcapsules of MOEO. The properties of the films were studied, and it was observed that they exhibited strong antioxidant activity [14].

To date, there has been no investigation into the impact of MOEO on the physiochemical characteristics of composite hydrogel-based films composed of pectin and collagen. Present study aims to investigate the effect of MOEO on the structural, mechanical, thermal, chemical, optical and barrier properties of hydrogel films based on pectin/collagen. In addition to investigating the physicochemical changes to determine the compatibility of the polymeric material with MOEO, the antioxidant activity was also evaluated as a potential food packaging material.

## 2. Results and Discussion

### 2.1. Visual Appearance

**Figure 1:**
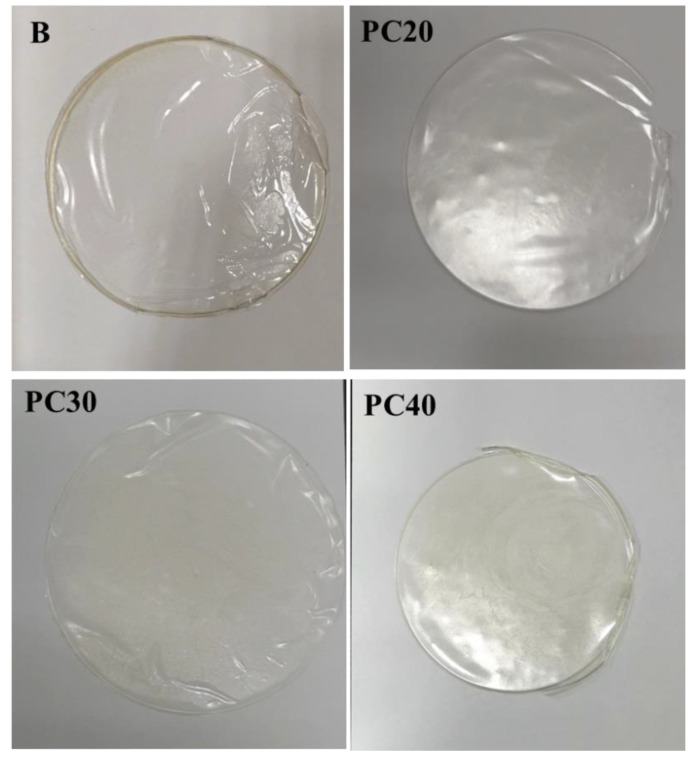
Visual appearance of B (control), PC20 (containing 0.1% MOEO), PC30 (containing 0.15% of MOEO), and PC40 (containing 0.2% MOEO).

The prepared edible film samples including B (Control), PC20, PC30, and PC40 were visually examined, and it was observed that control (B) and PC20 film samples had superior visual characteristics and was easy to peel from the Petri plate. Moreover, the PC20 film exhibited relatively improved characteristics regarding brittleness, flexibility, and stiffness. Film samples PC30 and PC40 containing 0.15% and 0.2% MOEO, respectively, were less transparent while exhibiting uniformity and flexibility. The better visual characteristics of the PC30 and PC40 film samples could be due to the addition of higher concentrations of MOEO and Tween 80, resulted in the uniform dispersion of the oil in the film matrix (Figure 1).

### 2.2. GC-MS Analysis of Melissa Essential Oil

**Figure 2:**
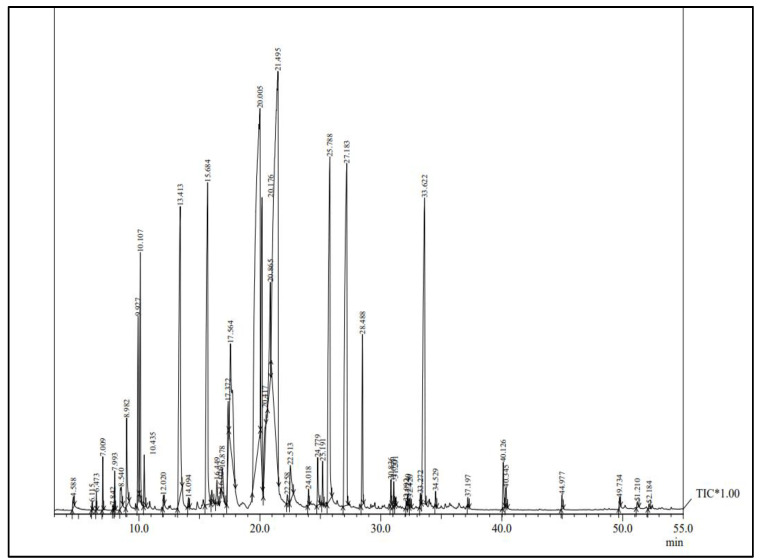
Chromatogram of Melissa essential oil.

The chromatogram of Melissa essential oil is shown in Figure 2. The main compounds detected in MOEO were 2,6-octadienal, 3,7-dimethyl (20.40%), citral (19.64%), caryophyllene (9.28%), geranyl acetate (7.33%), caryophyllene oxide (6.85%), citronellal (6.58%), and linalool (6.00%) (Table 1). As per Sorensen’s research [15], the primary constituents of *M. officinalis* obtained through laboratory distillation of verified plant material are the isomers of citral, geranial, and neral. Previous studies have reported that the essential oil of lemon balm contains citronellol and linalool as their primary chemical constituents [15,16]. Park and Lee [17] reported that the major compound of essential oil in lemon was 2,6-octadienal and 3,7-dimethyl-. The varied chemical components in the literature could be attributed to several factors influencing the chemical diversity of essential oils, such as light, precipitation, growth location, and soil [18].

### 2.3. Thickness

Table 2 presents the results that were obtained for the average thickness of the edible film samples. The thickness values varied between 0.061 and 0.073 mm by the incorporation of MOEO. Control film (B) had the lowest value of thickness (0.061 mm); however, the highest value (0.073 mm) was observed in PC40 with a maximum concentration (0.2%) of MOEO. The results demonstrate that incorporating MOEO in pectin–collagen-based hydrogel films had a significant (*p* > 0.05) effect on the resultant film thickness. The observed increase in thickness may be attributed to the incorporation of oil into the film matrix, which consequently led to the generation of microdroplets owing to the oil’s hydrophobic properties [19]. In our prior research, the incorporation of ginger essential oil had an impact on the thickness of hydrogel films composed of chitosan and porphyran [20].

### 2.4. Mechanical Attributes

The mechanical properties of biopolymer films are commonly evaluated through the measurement of TS and EAB. The mechanical resistance of a film is attributed to cohesive forces between chains, which is represented by its tensile strength. On the other hand, the flexibility of a film, or its capacity to elongate before breaking, is evaluated through its elongation at break. The concurrent study of tensile strength and elongation at break is necessary due to the structural characteristics of films, whereby those with high tensile strength typically exhibit low elongation at break [21].

Table 2 represents the values for the mechanical properties, including TS and EAB of pectin–collagen-based composite films loaded with MOEO. The study found that the tensile strength varied between 8.12 and 1.25MPa, with the control film exhibiting the highest value and PC40 exhibiting the lowest value. The values of elongation at the break exhibited significant variation (*p* < 0.05) ranging from 27.36 to 36.29%. The control film (B) demonstrated the lowest value, while the PC40 film showed the highest value. The incorporation of MOEO into the biopolymer network decreased TS caused mostly by intra- and inter-molecular interactions [13]. The observed enhancement in the EAB can be attributed to the incorporation of MOEO, which acted as a plasticizer and facilitated the mobility of polymer chains, thereby imparting flexibility to the films [22]. The results of the present study are consistent with those reported by Al-Harrasi et al. [20], wherein the incorporation of ginger essential oil into chitosan and porphyran-based composite film resulted in a reduction in tensile strength.

### 2.5. Barrier Properties

The water vapor permeability (WVP) and moisture content of hydrogel-based films made of pectin and collagen and loaded with different concentrations of MOEO were assessed. The results indicated that the control film sample (B) without the addition of MOEO showed higher values for WVP as compared to the film samples loaded with oil (Table 2). The WVP of the film samples decreased from 0.676 to 0.327 (g mm)/(m^2^ h kPa) with increasing concentrations of MOEO. This behavior of the films could be due to the hydrophobicity of the MOEO causing the inhibition of water permeability, since the Tween 80 has a hydrophilic character. The water WVP of the film could be affected by the hydrophilic–lipophilic ratio of the film matrix, as water diffuses through the hydrophilic section of the matrix [23]. In addition, the reduced WVP of the films could be ascribed to the crosslinking between the polymer chains and reduced chain mobility with the addition of MOEO and Tween 80, resulting from the filling of empty spaces in the film matrix [24]. Similar findings were reported by Almasi et al. [23], wherein the pectin-based hydrogel films resulted in to decrease in the permeability of water vapors when loaded with marjoram essential oil nanoemulsions.

Assessment of the moisture content is a crucial parameter because it affects the mechanical, barrier, and sensory characteristics of the films. The film samples that were loaded with MOEO exhibited a lower level of moisture content in comparison to the control film, as indicated in Table 2. The moisture content decreased from 29.92 to 25.16% with increasing concentrations of the oil in the film samples. The findings of the present investigation are consistent with those obtained by Nisar et al. [25], who found that the incorporation of clove bud essential oil into edible films based on citrus pectin resulted in a decrease in the amount of water present in the films.

### 2.6. Transparency and Color Analysis

The transparency of the edible films is an important parameter as it affects the visual appearance of the food products. The percentage transparency values varied significantly (*p* < 0.05) from 90.37 to 82.80% with the addition of oil in the films. The transparency of the control film (B) was highest, while the film sample, PC40 with the highest concentration of oil exhibited the lowest transparency. The reduction in transparency after the inclusion of MOEO may be attributed to the presence of colorful constituents within the oil [20]. The results of the current investigation are consistent with those reported by Scartazzini et al. [26], wherein a decrease in transparency was noted upon the incorporation of mint essential oil into films.

The color parameters of different film samples loaded with varying concentrations of MOEO are presented in Table 3. The lightness (L*) of the film samples slightly increased with higher MOEO concentration, but there were no significant differences observed in the values of the lightness of different samples. The inclusion of MOEO resulted in notable changes in the yellowness (b*) (0.95–1.57) of the film samples. The increase in the yellow color may be associated with the existence of various pigments within MOEO. Furthermore, a significant difference was detected in the a* redness values of the film samples. In addition, a higher ΔE* value was associated with a higher amount of MOEO; for instance, PC40 had the highest value of Δ*E* (1.90), and B film (control film) had the lowest value (0.85). These results demonstrated that the addition of MOEO changed the color characteristics of pectin-collagen-based composite films. Similar findings were reported by Al-Harrasi [20] et al., indicating that the incorporation of essential oil increased the yellowness of the edible films.

### 2.7. Scanning Electron Microscopy (SEM)

**Figure 3:**
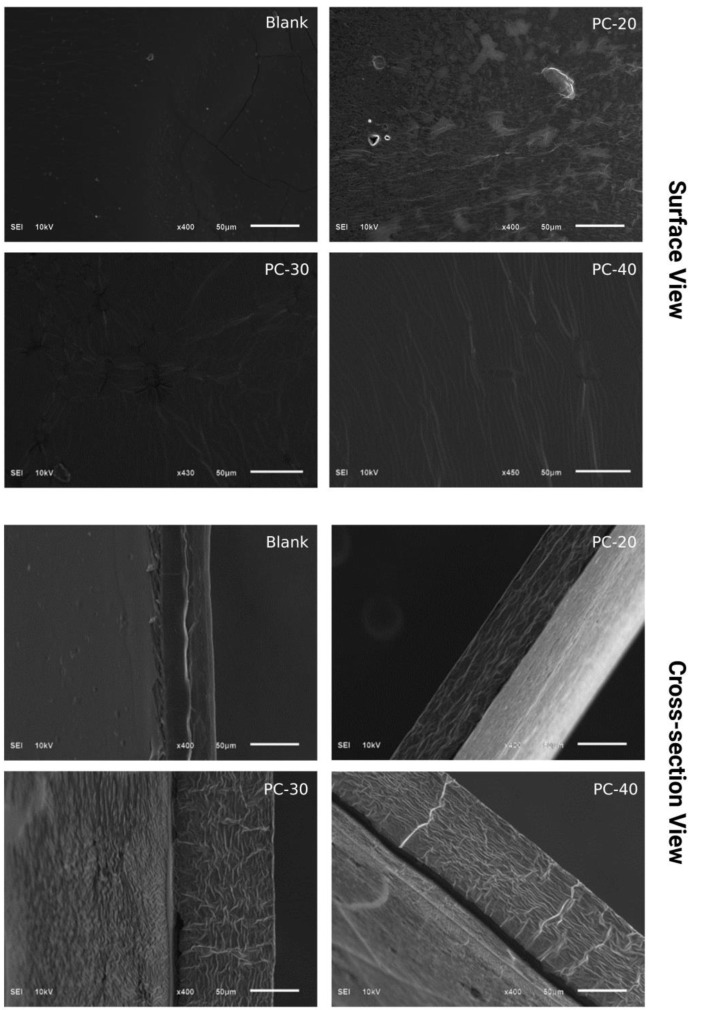
Morphological characteristics of B (control), PC20 (containing 0.1% MOEO), PC30 (containing 0.15% of MOEO), and PC40 (containing 0.2% MOEO).

SEM analysis can offer a comprehensive depiction of the surface texture of the film, which can help to determine its roughness, porosity, and other surface properties. SEM images of the prepared film samples with and without the addition of MOEO are presented in Figure 3. The surface morphology of the control film sample was observed as homogeneous, with cracks and microscopic particles all over the surface. PC20 film sample with 0.1% concentration of MOEO showed a rough surface with fewer cracks and particles across the surface. However, PC30 and PC40 film samples with the higher MOEO concentrations of 0.15 and 0.2%, relatively had homogeneous structures, and the oil was uniformly dispersed in the film matrix. Tween 80 stabilizes the oil-in-water emulsion, and the uniform dispersion of the oil in the film matrix could be due to the addition of the highest concentrations of Tween 80 in the PC 40 film sample. Overall, the SEM analysis revealed that films loaded with different concentrations of MOEO showed a more homogeneous structure with less numbers of particles, cracks, and pores as compared to the control films sample. The observed surface properties of the films may be attributed to the intermolecular interaction among the constituents that contribute to film formation, such as polymers, oil, and plasticizer.

### 2.8. X-ray Diffraction (XRD) Analysis

**Figure 4:**
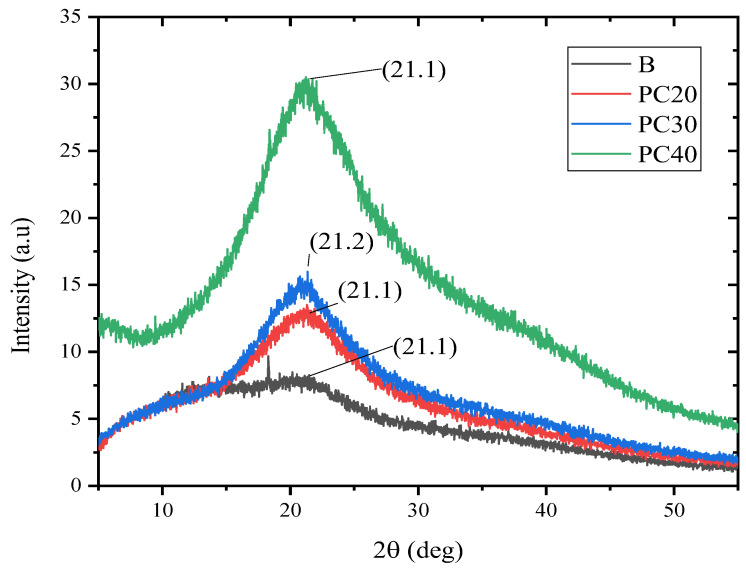
X-ray diffraction analysis of B (control), PC20 (containing 0.1% MOEO), PC30 (containing 0.15% of MOEO), and PC40 (containing 0.2% MOEO).

XRD analysis is an important tool for the characterization of edible films, as it can provide valuable information about their crystalline structure and properties, which is essential for their development and optimization. Figure 4 depicts an overlay of XRD diffraction peaks and intensities for different edible film samples loaded with MOEO. The XRD analysis indicated that the samples exhibit peaks at similar positions, although with differing intensities. The distinctive peaks of film samples loaded with MOEO and control were observed at position 21 of 2θ. It was observed that each of the film samples displayed a significant amorphous phase. The present research indicates that there is a reduction in the crystalline nature of the films as the concentration of MOEO increased. The tensile strength of edible film samples can be significantly influenced by their crystallinity. In general, a higher degree of crystallinity in the film leads to a higher tensile strength, while a more amorphous structure leads to a lower tensile strength.

### 2.9. Fourier Transform Infrared Spectroscopy (FTIR) Analysis

**Figure 5:**
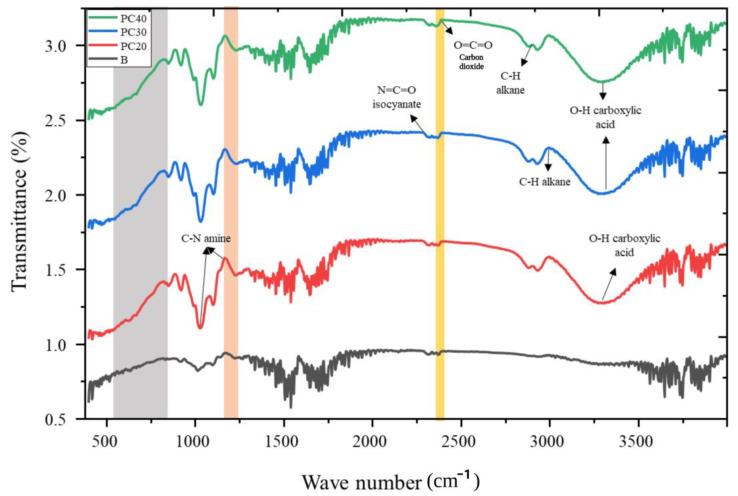
Fourier transform infrared patterns of B (control), PC20 (containing 0.1% MOEO), PC30 (containing 0.15% of MOEO), and PC40 (containing 0.2% MOEO).

FTIR analysis provides information about the interactions among functional groups of film constituents. The FTIR pattern of the film samples based on pectin and collagen containing different concentrations of MOEO is shown in Figure 5. The 3300 cm^−1^ region band peaks reflected OH stretching bonds. Inter-molecular or intramolecular hydrogen bonds express the stretching vibration of OH [13]. The O=C=O stretching vibrations were responsible for the peak at the 2389 cm^−1^ region. The active films containing MOEO exhibited bands in those areas, suggesting that MOEO was linked to the biopolymers comprising film matrix, as depicted in Figure 5 [27]. The band at 2274 cm^−1^ was linked to the N=C=O isocyanate group, whereas the band at 1170–1220 cm^−1^ was linked to the C–O stretching vibrations. The stretching vibrations of –OH and hydrogen bonding among the hydroxyl groups of the film-forming components can be indicated by a broad peak observed in the 3600–3200 cm^−1^ range [28]. Films loaded with MOEO showed an increase in the peak intensities of (–OH) stretching as compared to the control film sample, which indicates the formation of new hydrogen bonds with the addition of oil in the film matrix. Furthermore, alterations in the FTIR spectrum were noted in oil-loaded samples, resulting in the appearance of new characteristic peaks (Figure 5). In general, the FTIR examination demonstrated the interaction among the functional groups of the film-forming constituents.

### 2.10. Antioxidant Activities of the Hydrogel-Based Films

**Figure 6:**
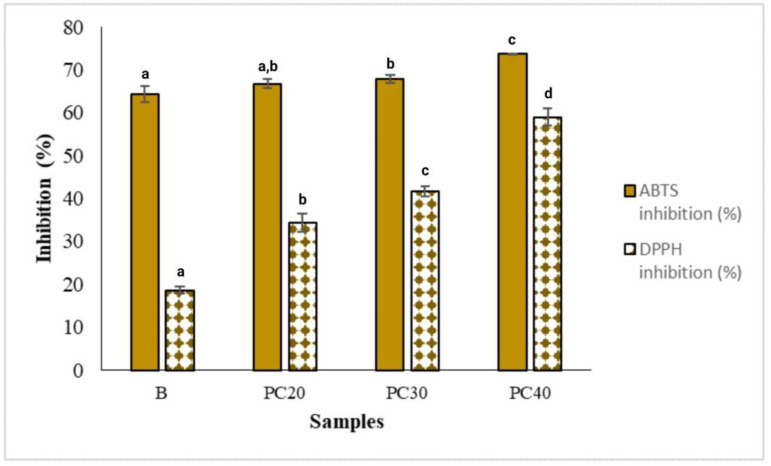
ABTS and DPPH inhibition percentage of B (control), PC20 (containing 0.1% MOEO), PC30 (containing 0.15% of MOEO), and PC40 (containing 0.2% MOEO). Different letters (a, b, c, and d) indicate significant differences (*p* < 0.05).

Edible films that contain essential oils or plant extracts exhibit promising antioxidant characteristics and offer a natural substitute for synthetic antioxidants. Additionally, these films enhance the sensory attributes, life expectancy, and overall quality of food products. The prepared hydrogel-based films containing MOEO were assessed for their antioxidant properties by using DPPH and ABTS assays. The results of the present investigation are shown in Figure 6, which indicate a rise in the DPPH and ABTS radical scavenging activity of the hydrogel-based films when loaded with MOEO. The highest DPPH and ABTS radical scavenging activity was shown by the PC40 film sample loaded with the highest concentration of MOEO; however, the lowest antioxidant activity was observed in the control film sample. The DPPH and ABTS radical scavenging activity in the film samples was enhanced with the addition of MOEO, increasing from 18.56 to 59.03% and from 64.31 to 73.75%, correspondingly. The improvement observed in the antioxidant activity of the films may be attributed to the existence of phenolic compounds in the MOEO.

Sani et al. [14] also reported similar results in which the sodium caseinate-based edible films possessed higher antioxidant activity when incorporated with a combination of zinc oxide nanoparticles (ZnONPs) and microcapsules of MOEO. Furthermore, many researchers have shown an increase in the antioxidant potency of edible films upon the incorporation of essential oils [28,29,30].

## 3. Conclusions

The *Melissa officinalis* essential oil (MOEO) appears as an interesting ingredient for the formulation of pectin and collagen composite-based hydrogel films. MOEO-loaded films showed more elasticity and thickness; however, the tensile strength and transparency were lower. The addition of the highest concentration (0.2%) of MOEO improved the barrier and antioxidant properties of the hydrogel-based films. The findings of the present investigation indicate that composite hydrogel films composed of pectin and collagen loaded with MOEO exhibit promising prospects as a viable food packaging material. However, more research is needed to enhance the mechanical and optical properties of the prepared films.

## 4. Materials and Methods

### 4.1. Material Procurement

Biopolymers including pectin and collagen with a purity of 95% were purchased from SRL Pvt. Ltd., Mumbai, India. The plasticizer (glycerol) was obtained from BDH Laboratory located in London, England. The Melissa oil used in this study was sourced from Nature Natural, a company based in Ghaziabad, India, and was identified by the batch number NNIMEEO/154/0821.

### 4.2. Film Preparation

The film-forming solutions of pectin (1.5% *w*/*v*) and collagen (1% *w*/*v*) were prepared separately by using distilled water. After the complete dissolution of polymers in distilled water, both solutions were blended. The solution was distributed in four labeled beakers (B, PC-20, PC-30, and PC-40), and concentrations of Tween 80, MOEO, and glycerol were added in the film-forming solutions, as shown in Table 4. The resultant solutions were eventually transferred into the respective Petri dishes and then subjected to drying at suitable room temperature conditions. Finally, the films were peeled and stored for further analysis.

### 4.3. Chromatographic (GC/MS) Analysis

Gas Chromatography–Mass Spectrometry (GCMS) analysis was performed to determine the composition of MOEO using GCMS-QP-2010 Plus Gas chromatograph Mass Spectrometer, Shimadzu, Japan. Sample size and GCMS conditions such as oven temperature, ramp rate, split ratio, the flow rate of Helium, and temperature of the ion source and injector were used as reported in our previous study [31]. The analysis was performed by similarity searches in the National Institute of Standards and Technology (NIST) mass spectra database with the obtained pure mass spectrum of each component.

### 4.4. The Thickness of the Films

The sample thickness (in mm) was determined by digital micrometer (Yu-Su 150, Yu-Su Tools, Shanghai, China). Five different measurements from different positions were obtained from each film sample, and the mean value was calculated.

### 4.5. Mechanical Testing

A texture analyzer (XT plus, Stable Micro Systems, Godalming, UK) was used to assess the mechanical strength of the films using the ASTM D882 standard method [32]. Tensile strength (MPa) and elongation at break (%) of the prepared samples were evaluated. The equations utilized in the determination of TS and EAB are as follows.

(1) $$TensileStrength(TS)=(\frac{F}{A})$$

The variable *F* denotes the maximum force, while *A* stands for the cross-sectional area of the film.

(2) $$Elongationatbreak(EAB)\left(\%\right)=\frac{Lf-Li}{Li}\times 100$$

The symbol *Li* represents the initial length of the film, while *Lf* denotes the length of the film in millimeters at the point of break.

### 4.6. Water Vapor Permeability (WVP)

The WVP of the film samples was determined by Erdem et al. [33]. The measured WVP of the films was calculated using Equation (3):

(3) $$WaterVaporPermeability=\frac{\Delta m}{\Delta t\times \Delta P\times A}\times d$$

The above equation involves the representation of ∆*m*/∆*t*, which denotes the amount of moisture gained per unit of time. The variable *A* represents the surface area of the film in square meters. The symbol ∆*P* denotes the disparity in water vapor pressure across the film, measured in kPa. Lastly, the letter *d* signifies the thickness of the film in millimeters.

### 4.7. Moisture Content (MC) 

The gravimetric method was used to determine the MC of the film samples by subjecting them to drying at 105 °C. The weight variation among films was determined using the following equation:

(4) $$MoistureContent=\frac{W1-W2}{W1}\times 100$$

where *W*1 and *W*2 represent the weight before and after drying of the films. 

### 4.8. Transparency and Color Analysis

The light transmission of the prepared films was quantified at 550 nm wavelength using an ONDA-Vis spectrophotometer, V-10 Plus, ONDA, Padova, Italy. Film strips were placed in a spectrophotometer test cell, and the transparency of the films was determined as per the methodology described by Zhao et al. [34].

A Konica Minolta colorimeter (Konica Minolta, Tokyo, Japan) was utilized to perform color analysis of the film samples. The L*, a*, and b* parameters were measured on a reference plate with a lightness value of 100. The analysis was conducted at different points on the surface of the films. The equation utilized for the computation of the overall color variation is as follows:

(5) $${\Delta E}^{*}={{\left[({\Delta L}^{*}\right)}^{2}+{\left({\Delta a}^{*}\right)}^{2}+{\left({\Delta b}^{*}\right)}^{2}]}^{1/2}$$

*L*: lightness, a*: green-red color, b*: blue-yellow color, and ΔE*: overall color variation.

### 4.9. Scanning Electron Microscopy (SEM)

The surface, as well as cross-sectional microstructural features of the films, was investigated using scanning electron microscopy (SEM) (JSM6510LA, Analytical SEM, Jeol, Tokyo, Japan) following the procedure as per our previous study [31].

### 4.10. X-ray Diffraction (XRD) Analysis

The percentage of crystallinity of the samples was recorded by an X-ray diffractometer (Bruker D8 Discover, Billerica, MA, USA) running with copper (Kα) radiation (1.5418 Å), 40 kV. Samples were analyzed between 2θ = 5° and 2θ = 50° at the rate of 0.500 s/point.

### 4.11. FTIR Spectra Analysis

The study employed an Infrared Tensor 37 instrument (InfraRed Bruker, Ettlingen, Germany) to evaluate the chemical interaction among the constituents of the film matrix. The Fourier Transform Infrared Spectroscopy (FTIR) instrument was configured to conduct 32 scans across a wide spectral region spanning from 400 to 4000 cm^−1^.

### 4.12. Antioxidant Activity

The free radical scavenging capability of the film samples was determined by using the DPPH and ABTS radical scavenging activity. The antioxidant activity of DPPH was studied as per the procedure described earlier by Brand-Williams et al. [35], utilizing 50 mg of film samples. The measurement of absorbance was conducted at a wavelength of 517 nm utilizing an ONDA Vis spectrophotometer. The outcomes were expressed as a percentage of inhibition. The antioxidant potential was assessed by using the chromogenic compound ABTS as per the procedure followed by Re et al. [36], with some minor modifications. The sample amount of 25 mg was used in the ABTS assay. The measurement of absorbance was conducted at a wavelength of 734 nm. The outcomes were expressed as a percentage of inhibition and were derived from an average of three measurements.

### 4.13. Statistical Analysis

The results were presented as mean ± SD of the triplicated determinations and were analyzed through a one-way analysis of variance using a statistical software package (SPSS ver. 17.0, SPSS Inc., Chicago, IL, USA). The current study presents results as the average and standard deviation (SD) of three separate measurements. The means were compared using Duncan’s test at a significant level of 5%.

## Figures and Tables

**Table 1:** Primary components detected in Melissa essential oil.

No.	NAME	R. Time	Composition %
1	2,6-OCTADIENAL,3,7-DIMETHYL	20.005	20.40
2	Citral	21.495	19.64
3	Caryophyllene	27.183	9.28
4	Geranyl acetate	25.788	7.33
5	Caryophyllene oxide	33.622	6.85
6	Citronellal	15.68	6.58
7	Linalool	13.413	6.00

**Table 2:** Thickness, mechanical, and barrier properties of edible film samples loaded with MOEO.

Film Samples	Thickness (mm)	Elongation at Break (%)	Tensile Strength (MPa)	Water Vapor Permeability (g mm/m^2^ h kPa)	Moisture Content (%)
B	0.061 ± 0.005 ^a^	27.36 ± 1.94 ^a^	8.12 ± 0.68 ^a^	0.676 ± 0.02 ^a^	29.92 ± 0.53 ^a^
PC20	0.068 ± 0.005 ^ab^	30.24 ± 2.23 ^bc^	3.48 ± 0.11 ^b^	0.569 ± 0.04 ^b^	28.30 ± 0.57 ^b^
PC30	0.072 ± 0.008 ^b^	34.97 ± 3.76 ^cd^	2.37 ± 0.21 ^c^	0.536± 0.05 ^b^	26.55 ± 0.23 ^c^
PC40	0.073 ± 0.006 ^b^	36.29 ± 2.80 ^d^	1.25 ± 0.16 ^d^	0.327 ± 0.01 ^c^	25.16 ± 0.83 ^c^

The values with different letters (a, b, c, and d) inside a column indicate significant differences (*p* < 0.05).

**Table 3:** Color parameters of different edible film samples loaded with MOEO.

Film Samples	Transparency (%)	*L*	a*	b*	ΔE*
B	90.37 ± 0.55 ^a^	95.82 ± 0.05 ^a^	−0.10 ± 0.01 ^a^	0.95 ± 0.07 ^a^	0.85 ± 0.07 ^a^
PC20	87.30 ± 0.28 ^b^	96.07 ± 0.02 ^a^	−0.15 ± 0.02 ^b^	1.32 ± 0.13 ^b^	1.16 ± 0.08 ^b^
PC30	84.33 ± 0.55 ^c^	96.52 ± 0.34 ^a^	−0.17 ± 0.01 ^b^	1.57 ± 0.27 ^bc^	1.63 ± 0.01 ^c^
PC40	82.80 ± 0.75 ^c^	96.54 ± 0.46 ^a^	−0.23 ± 0.02 ^c^	1.57 ± 0.06 ^c^	1.90 ± 0.01 ^d^

The values with different letters (a, b, c, and d) inside a column indicate significant differences (*p* < 0.05). *L*: lightness, a*: green-red color, b*: blue-yellow color, and ΔE*: overall color variation.

**Table 4:** The composition of the film-forming solution for different samples.

Film Samples	Film Forming Components
B/Control	(1.5%) Pectin (*w*/*v*) + 1% Collagen (*w*/*v*) + (1.25%) Glycerol (*v*/*v*)
PC20	(1.5%) Pectin (*w*/*v*) + 1% Collagen (*w*/*v*) + (1.25%) Glycerol (*v*/*v*) + (0.2%) Tween 80 (*v*/*v*) + (0.1%) MOEO (*v*/*v*)
PC30	(1.5%) Pectin (*w*/*v*) + 1% Collagen (*w*/*v*) + (1.25%) Glycerol (*v*/*v*) + (0.3%) Tween 80 (*v*/*v*) + (0.15%) MOEO (*v*/*v*)
PC40	(1.5%) Pectin (*w*/*v*) + 1% Collagen (*w*/*v*) + (1.25%) Glycerol (*v*/*v*) + (0.4%) Tween 80 (*v*/*v*) + (0.2%) MOEO (*v*/*v*)

## Data Availability

Not applicable.
